# 

**Published:** 1904-03-12

**Authors:** 


					?ujZ hospital,. "The standard of highest purity."?Lancet. .arch 12th, 1904
OADBURY'S rt- 'W 'Irs CADBURY'S
COCOA
"A PERFECT FOOD."
COCOA
NO DRUGS OR ALKALIES USED.
NO. 911. Yol.XXXY. [afeSfpen] SATURDAY,
March 12th, 1904.
[subs?ripttonTernis,] pR|CE JHREEPENCE.

				

